# Experimental and Computational Analysis of Synthesis Conditions of Hybrid Nanoflowers for Lipase Immobilization

**DOI:** 10.3390/molecules29030628

**Published:** 2024-01-29

**Authors:** Danivia Endi S. Souza, Lucas M. F. Santos, João P. A. Freitas, Lays C. de Almeida, Jefferson C. B. Santos, Ranyere Lucena de Souza, Matheus M. Pereira, Álvaro S. Lima, Cleide M. F. Soares

**Affiliations:** 1 Postgraduate Program Process Engineering, Tiradentes University (UNIT), Campus Farolandia, Aracaju 49032-490, Sergipe, Brazil; danivia.endi@souunit.com.br (D.E.S.S.); lays-carvalho@hotmail.com (L.C.d.A.); jeffersoncleriston5@gmail.com (J.C.B.S.); ranyerels@hotmail.com (R.L.d.S.); 2 Institute of Technology and Research (ITP), Aracaju 49032-490, Sergipe, Brazil; 3 Department of Chemical Engineering, University of Coimbra, CIEPQPF, 3030-790 Coimbra, Portugal; 4 Postgraduate Program Chemical Engineering, Federal University of Bahia (UFBA), Campus Federação, Salvador 40210-630, Bahia, Brazil; aslima2001@yahoo.com.br

**Keywords:** lipase hybrid nanoflowers, electrostatic interactions, enzyme protonation

## Abstract

This work presents a framework for evaluating hybrid nanoflowers using *Burkholderia cepacia* lipase. It was expanded on previous findings by testing lipase hybrid nanoflowers (hNF-lipase) formation over a wide range of pH values (5–9) and buffer concentrations (10–100 mM). The free enzyme activity was compared with that of hNF-lipase. The analysis, performed by molecular docking, described the effect of lipase interaction with copper ions. The morphological characterization of hNF-lipase was performed using scanning electron microscopy. Fourier Transform Infrared Spectroscopy performed the physical–chemical characterization. The results show that all hNF-lipase activity presented values higher than that of the free enzyme. Activity is higher at pH 7.4 and has the highest buffer concentration of 100 mM. Molecular docking analysis has been used to understand the effect of enzyme protonation on hNF-lipase formation and identify the main the main binding sites of the enzyme with copper ions. The hNF-lipase nanostructures show the shape of flowers in their micrographs from pH 6 to 8. The spectra of the nanoflowers present peaks typical of the amide regions I and II, current in lipase, and areas with P–O vibrations, confirming the presence of the phosphate group. Therefore, hNF-lipase is an efficient biocatalyst with increased catalytic activity, good nanostructure formation, and improved stability.

## 1. Introduction

The behavior of an immobilized enzyme is influenced primarily by its support, therefore, incorporating new technologies through support capable of enabling bioprocesses may be able to overcome some of the limitations [1,2,3]. Immobilized enzymes should exhibit specific characteristics, including high enzymatic activity, a large surface area, and operational stability. The properties of nanostructured complexes, which are effective for enzyme immobilization, provide increased catalytic activity, stability, surface area, and the possibility of reusing enzymes several times [4,5,6].

The methods frequently used to immobilize lipases exhibit limitations, particularly a decrease in catalytic activity, which directly affects the enzyme performance in the reaction [7]. Developing lipase hybrid nanoflowers (hNF-lipase) is innovative for enzymatic immobilization, as it can generate biocatalysts that overcome the existing limitations of more conventional methods. The differential of this structure lies in the synthesis mechanism that immobilizes the enzyme through nucleation, coordination, precipitation, self-assembly, and growth. Studies report that bivalent metal ions, such as the Cu (II) chosen, are efficient for precipitation. Furthermore, a phosphate-buffered solution is commonly used in different protocols to form hNF-lipase effectively [5,8,9].

In nucleation, electrostatic forces cause copper ions to bind to phosphate, forming primary crystals. In the coordination, nitrogen molecules coordinate these crystals to the enzyme surface [3,10,11,12]. In this process, the precipitation of petal-like nanopetals occurs. Finally, the lipase–metal ion–buffer concentration complex molecules bind in self-assembly process. During the incubation period, the growth and consequent stabilization of the biocatalyst occur [13]. The reaction results from the coordination and precipitation effect of the anions and cations in the nanoparticles, thus forming organic–inorganic complexes. Hybrid nanoflowers composed of inorganic metal ions/clusters and organic linkers have attracted much attention owing to their designable structures and adjustable properties [6,14].

The technique is still relatively new, and the factors influencing the interactions between lipase–metal–phosphate nanoflowers have not yet been well described [9]. For example, most studies on the formation of nanoflowers have focused on using cupric ions. Still, there is little evidence of the other interactions between bivalent metal ions and enzyme amino acids. In addition, lipase applications in nanoflowers structures have shown high enzymatic activity due to the level of interfacial activation in organic media [13,15,16].

This enzyme enables it to catalyze ester hydrolysis reactions, esterification, and transesterification to generate an ester [17,18]. Lipases are promising enzymes in the protocol for obtaining hNF-lipase for application in the industry to the produce of biodiesel and biofuels [2,5,19,20]. Li et al. [3], Li et al. [16], and Ren et al. [21] described works in the literature that influenced the concentrations of the selected buffers, ranging between 0.1 mM and 10 mM. Escobar et al. [22] utilized a concentration of 50 Mm, while Cui et al. [6] used 100 mM. Ke et al. [23] performed the immobilization of *Burkholderia cepacia* (BCL) on the nanoflower structure and evaluated various buffer concentrations, from 5 to 30 mM.

Liu et al. [14] and Jiang et al. [19] studies report used lipases and copper ions to synthesize hybrid nanoflowers. They confirmed the increase in catalytic activity, flower conformation, and stability by visualizing their nanoflower structure. However, these studies require more computational analyses, such as evaluating molecular dynamics, which could elucidate the mechanism of interactions in the synthesis of hNF-lipase. Computational analysis may also predict possible interactions between lipase and phosphate metals and justify the experimental results.

There are no more comprehensive studies on the influence of current interactions on the formation of hybrid lipase nanoflowers (hNF-lipase) across buffer concentrations and pH values. Literature studies have reported the influence of pH on nanoflowers [22,24,25,26]. In this sense, the reactions utilized different pH values (5–9) and concentrations of phosphate buffer (10–100 mM) to evaluate the interaction for obtaining the nanoflower. The computational analysis performed for various pH values explains the data. Thus, the enzymatic activity and electrostatic interaction data were analyzed based on the formation of nanoflowers created under different synthesis conditions. These data can confirm the nanostructures in the conformation of flowers.

This study evaluated the formation of hybrid lipase nanoflowers (hNF-lipase) using experimental and computational analyses under different synthesis conditions. The innovation in this investigation relates to an increase in the activity of hNF-lipase regarding free lipase because of the ionic strength of the buffer in coordinating the ions in the formation mechanism and the increase in the surface area of the nanoflower. The innovation differential of this work is to use molecular docking to describe the coupling interaction between the metal ion and the enzymatic structure to interpret the types of molecular interactions that promote the formation of hNF-lipase.

In this sense, the reactions utilized different pH values and phosphate buffer concentrations to evaluate their combined influence on obtaining the nanoflower. Thus, an analysis of the variation of elaborated nanoflowers for enzymatic activity and operational stability data. Finally, a significant innovation in this work is using molecular docking to describe the coupling interaction between the metal ion and the enzymatic structure, providing insights into the types of molecular interactions that promote the formation of hNF-lipase.

Therefore, this aims of this work is the investigation of lipase hybrid nanoflowers, which are relevant for development innovative technologies for biocatalysis. The evaluation of nanostructure synergism seeks to understand the catalytic potential through experimental data and molecular docking. The purpose of this study is to form a protocol carrying out immobilization using copper phosphate crystals for the mechanism of the hNF formation protocol with commercial lipase from *Burkholderia cepacia* in various pH values and concentrations of a phosphate-buffered solution (PBS).

## 2. Results and Discussion

### 2.1. Activity of hNF-Lipase

**Figure 1:**
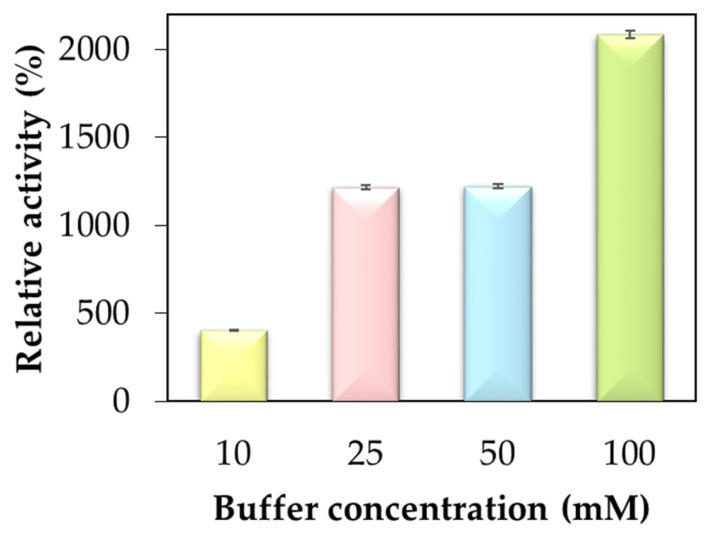
Relative activity of hNF-lipases relative to the free enzyme, using pNFA as the substrate, at pH 7.4, for different buffer concentration (10–100 mM) (*p* < 0.01).

The enzymatic activity of hybrid nanoflowers was determined using p-nitrophenyl acetate (pNFA) as a substrate. Initially, studies conducted with nanoflowers used pH 7.4 at different buffer concentrations (10–100 mM). The results in Table 1 present the hydrolytic activity values of free lipase and immobilized biocatalysts in the studied variations. Free enzymes showed activities of 42.62 U g^−1^ in different buffer concentrations (*p* < 0.01). Figure 1 shows that an increase in catalytic activity follows an increase in phosphate concentration. The highest value of relative activity of 2085%, or approximately 20 times, was obtained at the maximum buffer concentration of 100 mM. The superior catalytic performances occur to the conformational modulation and the hierarchical structure of hNF-lipase. This increased concentration of anions and cations causes more molecular interactions between the compounds [27].

**Figure 2:**
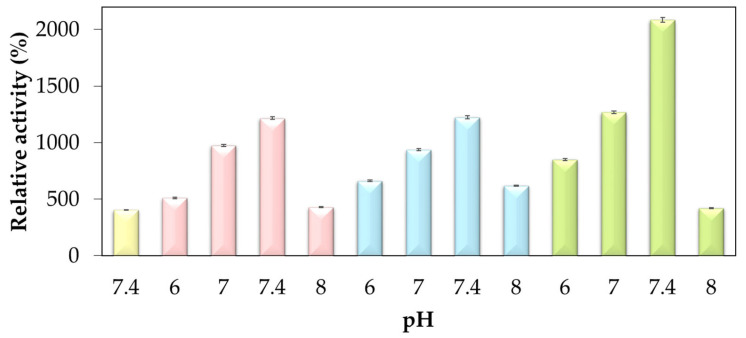
The activity of nanoflowers concerning the free enzyme (yellow), with pNFA as the substrate. Different concentrations of buffer concentration, 25 mM (pink), 50 mM (blue), and 100 mM (green), at different pHs (6–8) (*p* < 0.01).

Thus, to expand the study on the influence of anions and cations on the activity of the biocatalyst, the protocol was carried out for different pH values, 6, 7, 7.4, and 8, in various concentrations of phosphate buffer (10, 25, 50, and 100 mM), as shown in Figure 2. However, no precipitate formed at pH 5 and 9, resulting in no formation of hNF-lipase. This finding was attributed to the repulsion of positive or negative charges in the presence of more acidic or more basic pH values, respectively, which causes the denaturation of the enzyme. The lowest concentration, 10 mM, resulted in the lowest percentage of activity, namely 403% at pH 7.4 (Figure 1).

Low activity of hNF-lipase was observed at a low phosphate radical concentration (<10 mM). With increasing phosphate radical concentration (>50 mM), the activity of hNF-lipase increased. The most significant activity level was obtained at the highest concentration of the phosphate radical of 100 mM. These data confirm the studies by Li et al. [8] concerning increased concentrations of phosphate radicals for other lipases and metal ions (Ag^3+^, Ca^2+^, Mg^2+^, Zn^2+^, Mn^2+^, Fe^3+^, and Al^3+^) [8]. These results demonstrate the positive effect of modifying the nucleation points in the protein structure on lipase modification, even if the nanoflower structure cannot be produced. This study suggested that part of the benefits of enzyme immobilization using nanoflowers is the modification of the enzyme groups, and not just the global coating of the enzyme surface of the metal shell [8,28].

In this way, this may have general applicability to other immobilized enzymes, with the advantage of being able to select the mechanical properties of the final biocatalyst. Thus, this study seeks to evaluate the buffer concentration effect and the immobilization protocol dependence of the applied on the lipase function at pH 7.4 (Figure 1).

As shown in Figure 2, it was also possible to verify a significant increase in the activity of hNF-lipase about free lipase, when using pH 7, in a buffer concentration of 100 mM, with the value of this increase being 1267%. Activity increase values of 1215% and 1223% were also found for pH 7.4 with 25 and 50 mM buffer concentrations, respectively. Thus, pH 7.4 is the most favorable for increased activity. Furthermore, the enzymatic activities at pH 7 with 25 and 50 mM buffer concentrations, and pH 6 with 100 mM, showed significant values (>1000%). A justification for the data found could be based on the proportion of the number of anions and cations present in these variations directly influencing the increasing in the activity, showing that the enzyme is interacting more and more with the inorganic part present.

Lisboa et al. [29] report that a neutral pH is beneficial for immobilized BCL activity, with the maximum relative activity reached between pH 6 and 8. According to Carvalho et al. [30], for *Burkholderia cepacia* free lipase, the optimal pH is pH 7, which may explain the higher activity values in this range. Similar behavior of hNF-lipase activity was obtained by Li et al. [8], in which higher relative activities were obtained at pH values above 7, while for pH 6 a low level of activity of hNF-lipase was observed for hNF-lipase Ca_3_(PO_4_).

The most relevant value of the enzymatic activity was obtained with the nanoflower pH 7.4 in the highest concentration of the buffer (100 mM) (Figure 1). Therefore, when analyzing buffer concentrations at different pHs, the best result occurs at pH 7.4 (Figure 2). The data show that more basic or more acidic pH values end up negatively influencing the enzymatic activity, which finds its best values at more neutral pHs (pH 7 and 7.4). Also, at the highest buffer concentration, lipase is more likely to precipitate along with the ions from the solution.

The literature reports increased catalytic activity of enzymes immobilized in the hNF-lipase [13,16,24,31]. Activity values have also been defined in units of p-nitrophenol produced (U). Li et al. [16] presented data the activity in BCL of 874.5 U g^−1^, and the protocol was performed with phosphate buffer and copper metal ions (120 Mm) at pH 7.4. The present work presented, at pH 7.4 (100 mM), enzymatic activity values of 888.43 U g^−1^, confirming the literature data for thus presenting activity data similar to BCL–phosphate–copper.

The improved activity demonstrates the synergistic effect by demonstrating the effect of energetic charges on structuring the enzymatic reaction. Furthermore, hNF-lipase has higher catalytic activity than free lipase because of the ionic strength of the buffer in coordinating the ions in the formation mechanism and the increase in the surface area of the nanoflower. The data provide evidence of increased enzymatic activity when inserted into the nanostructure. It also demonstrates that changing the synthesis conditions may directly influence the enzymatic activity of the nanoflower. Furthermore, the percentage activity values are higher when the pH values are more neutral. Therefore, the molecular distribution and interactions in the hNF-lipase structure warrant further study.

### 2.2. Computational Analysis

The effect of pH on the formation of hNF-lipase was experimentally evaluated, showing that below pH 6 and above pH 8 hNF-lipase were not formed. protonation calculations on the BCL crystal structure were performed to identify the role of amino acids on the charge of BCL. BCL is a lipase composed of 320 amino acids and displays an isoelectric point at pH 6.3 [32,33]. Therefore, BCL residue charges are expected to be primarily positive at an acidic pH and the opposite at an alkaline pH.

**Figure 3:**
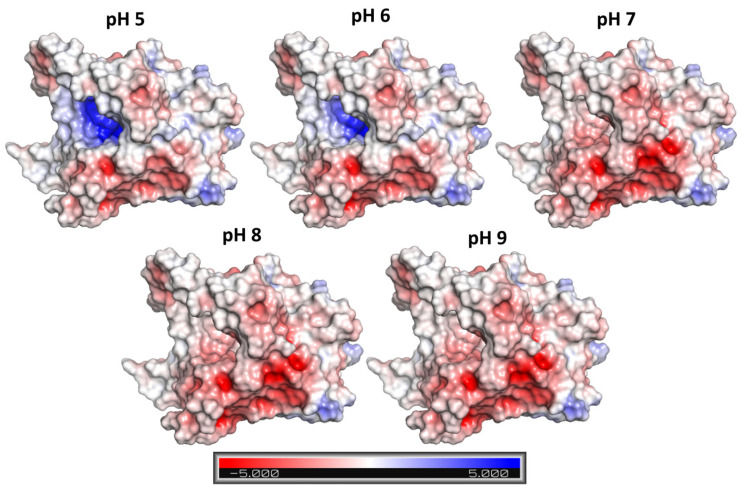
Electrostatic charge at the BCL surface, calculated for pHs 5 to 9. Red–white–blue scale refers to minimum (−5 kT/e, red) and maximum (5 kT/e, blue) surface potential.

Figure 3 shows the electrostatic charge at the BCL surface in the pH range from 5 to 9. At the most acidic pH studied (pH 5), the amount of positively charged amino acids is noticeable (blue surface in Figure 3). With the increase to pH 6, there is a decrease in positively charged amino acids and a consequent decrease in the blue area on the enzyme surface. From pH 7 onwards, the positive charges begin to show a neutral charge, highlighting the role of negative charges on the enzyme surface. At pH 8 and 9, the charge of the amino acids on the surface appears to remain similar. These results suggest that excess positive or negative charges affect the interaction with the structure.

According to the results obtained, the possible effect of protonation on the formation of hNF-lipase occurs through copper sulphate ions the attraction and repulsion capacity. At an acidic pH, the positively charged amino acids, namely tyrosine, arginine, lysine, glutamic acid, and histidine, promote the repulsion of copper ions (Cu^2+^) and, consequently, inhibit the formation of hNF-lipase. On the other hand, at an alkaline pH, the absence of positive charges potentiates the repulsion capacity of the sulphate ions (SO_4_^2−^) present in metal sulphate nanocrystals.

Therefore, the pH range between 6 and 8 (slightly below and above the BCL isoelectric point) seems to be ideal for the formatting hNF-lipase using BCL. In fact, when evaluating the effect of pH on the formation of hNF using copper sulfate and the enzyme lactoperoxidase (LPO), Altinkaynak et al. [34] found the same behavior evidenced here. LPO has an isoelectric point of approximately 9. Therefore, at an acidic pH (pH 4 and 5) hNF-lipase structures were not formed.

**Figure 4:**
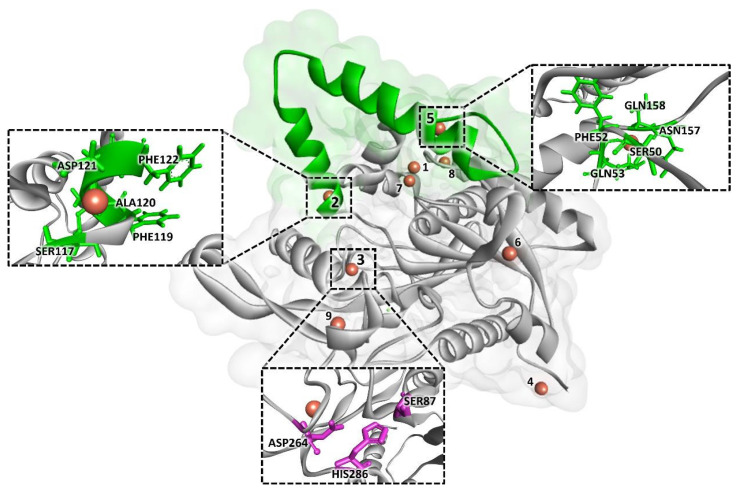
All molecular docking poses of BCL with Cu^2+^. Lid domain in green and catalytic triad amino acids (SER87, ASP264, and HIS286) in purple.

Subsequently, molecular docking analysis was performed to identify the binding sites and the specific interactions between the copper ion (Cu^2+^) and the BCL amino acids. Molecular docking analysis was performed with BCL protonated at pH 7.4 (pH value with higher enzymatic activity found through experimental evaluation). All anchoring positions between Cu^2+^ and BCL amino acids and, the type of interaction, are shown in Table S1 (Supplementary Material). According to the results obtained, Cu^2+^ ions show in eight of the nine binding site-identified van der Waals interactions with BCL amino acids. Figure 4 shows all Cu^2+^ binding sites in the BCL structure, emphasizing the poses of the binding sites (2, 3, and 5) identified in regions that may be directly linked to the final performance of the biocatalyst.

The molecular interaction diagrams of the docking poses are displayed in Figure S1, in the Supplementary Materials. At the highest values of the docking score (>1), van der Waals interactions occur with the amino acids: SER54, ASP55, ASP56, GLN88, SER117, PHE 119, ALA120, ASP121, PHE122, and THR 166. At the intermediate docking scores (between 1 and 0.5), the amino acids TYR4, ASP2, ASN3, SER50, PHE52, GLN53, ASN157, GLN158, GLY211, ASN263, ASP264, GLY265, VAL267, and SER268 interact through van der Waals interactions. Finally, at the lowest docking score (lower than 0.5), van der Waals interactions were identified with ASP21, GLN34, VAL46, ASP55, ASP56, LEU91, VAL 99, and THR166.

Additionally, in addition to the types of interactions and amino acid residues involved, according to the literature, the positioning of the binding sites in relation to important structures in the enzyme can also influence the improvement of the activity [22]. The authors report that in their studies with lipase from *Thermomyces lanuginosus* and β-galactosidase, respectively, the distance of the metal ion binding sites from the ends of the enzyme lid, and/or the region of the active site, favors the performance of the enzymes. In the present study, sites 2 and 5 are located at the ends of the enzyme lid 118–159 [35], and site 3 is close to the BCL active site region (SER87, ASP264, and HIS286 [36]. However, an increase in the catalytic activity of hNF-lipase was identified, compared to the free enzyme, indicating that the presence of Cu^2+^ ions in these regions of the BCL probably favors catalytic efficiency due to the active conformation of the lipase in hNF-lipase.

### 2.3. Morphology of hNF-Lipase

**Figure 5:**
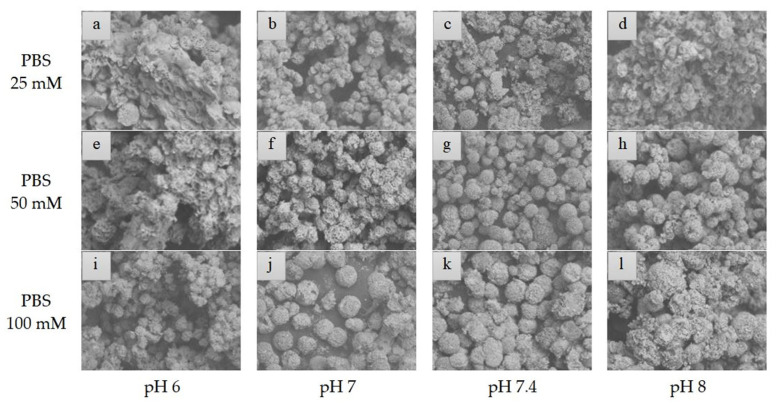
SEM images of hNF-lipases at different pHs (6–8) and at different buffer concentrations: (**a**–**d**) 25 mM, (**e**–**h**) 50 mM, and (**i**–**l**) 100 mM.

The variations in the morphology of organic–inorganic nanoflowers were analyzed used scanning electron microscope (SEM). The micrographs showed the formation of nanoflowers under the different conditions of the protocol, thus indicating that the enzyme is stabilized within the hNF-lipase structure (Figure 5). The variation in the morphology of the micrographs was observed at different concentrations of buffer (10–100 mM) and pH (6–8). After the growth period, all samples showed spherical and open shapes. Compared to the literature, the nanoflowers produced have a similar appearance to the reported nanoflowers, although there are differences between the synthesis variations [16,21].

From the comparison of the different micrographs presented, it was possible to affirm that pH 6 presented malformed nanoflowers in all buffer concentrations. The formed nanoflowers showed more compact structures at more neutral pH values [22]. Figure 5 shows that hNF-lipase has a larger flower-shaped conformation at pH 7.4, and 8, and 100 mM buffer concentration (Figure 5i–l). However, for pH 8, the diameter of the formation increases as the concentration of the buffer increases, presenting amorphous nanoflowers at the highest concentration; this result can be explained by a saturation of the charges denaturing the enzyme. The most clearly well-formed nanoflower can be observed at pH 7.4, with a buffer concentration of 100 mM. These data evidenced the enzyme activity values found in a previous topic.

The micrographs show that at the highest buffer concentration, these nanostructures appear to cluster together. At 25 mM (Figure 5a–d), it is observed that the nanoflowers are more dispersed and more distant from each other for all the pHs, in addition to showing deformations. The results show that the nanoflowers were not well developed at the lowest concentrations of phosphate buffer and pH values. Consequently, the nanoflowers present in the phosphate buffer at a concentration above 50 mM have more developed structures, especially at higher pHs, as explained in the literature (pH 7, 7.4, and 8) [23,25]. Therefore, the higher buffer the concentration, the higher the density of the petals in the nanoflowers in this analysis.

Thus, we can verify that relating to the crystals of Cu_3_(PO_4_)_2.5_H_2_O, as described in the literature, the nanoflowers showed differences in the density of the petals and hindered the mass transfer of the nanoflower at a higher pH and that for neutral pH values, there is increased activity [1,8,37]. In this study, the best results were obtained with 100 mM, indicating the need for this type of evaluation, and at pH 8 with 100 mM of buffer concentration, the formation seems to deform due to more excellent saturation of the precipitates. This phenomenon is due to more anions in the salts present, which causes an exaggerated agglomeration effect and the consequent deformation of the particles. It is believed that the saturation of the compounds in the solution directly affects the precipitation of the nanoplates. Nanoflowers formed at pH 7.4 visibly show the most consistent and stable structures at different concentrations of buffer concentration, with the best result being found in hNF-lipase with 100 mM (Figure 5i–l).

From these data, the variation in the nanoflowers needs to consider all the changes in the anions and cations. Therefore, the conformational mobility of nanoflowers is subject to changes in the aqueous medium that are responsible for the decrease or increase in the catalytic activity, as influenced by the pH and buffer concentration. The strategy in this study was to use experimental and in silico analysis to evaluate hNF-lipase morphology; according to the results obtained, protonated BCL at pH 7.4 has all the anchoring positions between the Cu^2+^ and BCL amino acids, and the type of interaction, and possibly through morphological analysis, the petal density of nanoflowers is ideal and exhibits the highest enzymatic activities compared to the native enzyme (~20 times). The experimental and computational analysis can still be complemented by an evaluation of the chemical structure of the hNF-lipase using the FTIR spectrum.

### 2.4. FTIR of hNF-Lipase

**Figure 6:**
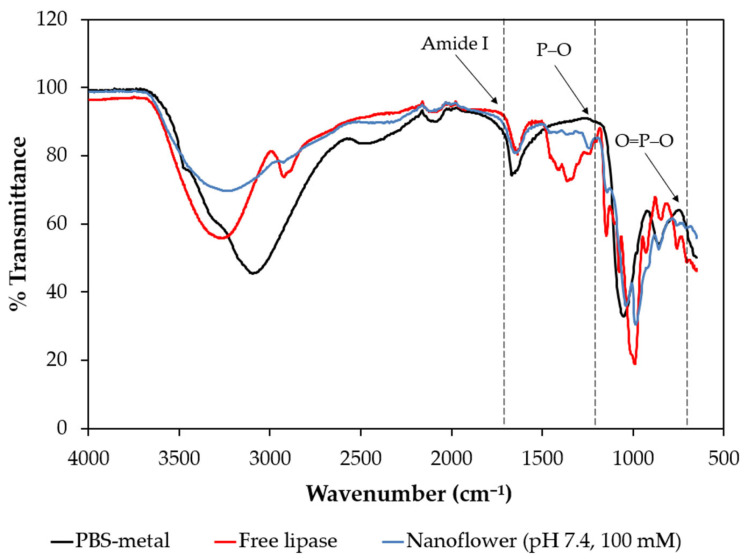
FTIR spectrum for hNF-lipase synthesized at pH 7.4 (100 mM) compared with free lipase and PBS-Cu^2+^.

The chemical structures of hNF-lipase were monitored using FTIR. The observation of the FTIR spectrum showed peaks at different wavenumbers (Figure 6). Peaks ranging from 3500–2800 (cm^−1^) were observed, which are characteristic of the presence of -CH_2_ and -CH_3_. The peaks present in the region of 1700–1600 (cm^−1^) and 1600–1500 (cm^−1^) are typical of lipase absorption exclusive to the protein secondary structure, which is present for amide I (C=O) and amide II (N–H and C–N), respectively. The analysis indicated the presence of lipase in the nanoflowers, as described in the literature, for the peaks present in the same region for different nanoflowers [11,18,38].

In the region of 1200–950 (cm^−1^), there is a robust characteristic peak that can be attributed to vibrations and elongations of P–O, found in the presence of phosphate groups [12,21,38]. The relatively small band at 530–670 (cm^−1^) was likely the phosphorus bridge bending vibrations, such as O=P–O, contributing to the phosphate groups [25,39]. Characteristic peaks are found due to the PO_4_^−3^ vibration, evidencing the existence of PBS-Cu^2+^ in the analyzed nanoparticle (Figure 6).

## 3. Material and Methods

### 3.1. Materials

This work used Amano lipase from *Burkholderia cepacia* (40 U g^−1^, pH 7, 50 °C) purchased from Sigma-Aldrich Co. (Saint Louis, MO, USA). The salts used to obtain the phosphate buffer, Na_2_HPO_4_ and KH_2_PO_4_, were obtained from Neon (São Paulo, SP, Brazil). Cupric sulfate 5-hydrate (98.5%) was obtained from Vetec (Rio de Janeiro, Brazil). The substrate used to evaluate the activity was p-nitrophenyl acetate (pNFA) 99%, obtained from Sigma-Aldrich Co. (Saint Louis, MO, USA), with Triton X and Isopropanol obtained from Dinâmica Química Contemporânea (São Paulo, SP, Brazil). A bovine serum albumin solution obtained from Sigma-Aldrich Co. (Saint Louis, MO, USA) was used to verify the protein concentration.

### 3.2. Synthesis of Hybrid Nanoflowers Lipase

The synthesis of hNF-lipase was based on the studies by Ge et al. [9] and Sharma et al. [12], with modifications. In the protocol performed, the enzyme solution was made with the addition of 5 mg of lipase mL^−1^ of phosphate-buffered solution. The saline solution was prepared by dissolving CuSO_4.5_H_2_O to obtain a concentration of 120 mM of metal ions. Then, the saline solution was added to the enzyme solution, to achieve a concentration of 1.2 mM of metal ions in the final solution.

The mixture was incubated for 72 h at room temperature (25 ± 5 °C). After incubation, it was possible to visualize the blue precipitate at the bottom of the reaction tube, thus allowing the removal of the supernatant. Finally, the precipitates were deposited on a glass plate and dried for 24 h at room temperature, after which scraping was performed to recover the mass of the biocatalyst. In order to evaluate the best condition for hNF-lipase synthesis, different pHs (5, 6, 7, 7.4, 8, and 9) and phosphate buffer concentrations (10, 25, 50, and 100 mM) were tested for the protocol. Therefore, the aliquots were separated and placed on a multiplate to determine the protein concentration and enzymatic activity of hNF-lipase. In addition, hNF-lipase was stored under refrigeration at a temperature of 4 °C.

### 3.3. Quantification of Total Proteins

The protein content of the lipases was determined using the [40]. From the standard curve prepared with bovine serum albumin (BSA) in distilled water, the absorbance of the sample was read using an Epoch 2 Microplate multiplate spectrophotometer (BioTek Instruments, Winooski, VT, USA) at a wavelength of 595 nm. The Bradford method defines immobilized lipase by comparing the enzyme solution before and after immobilization.

To determine the BCL protein content, an aliquot of 50 μL was removed from the enzymatic solution, which was deposited in the multiplate cuvette and mixed with 150 μL of Bradford’s solution and shaken for 1 min. After 15 min the absorbance reading was performed. The absorbance values were converted into concentration from the previously prepared calibration curve. From the data obtained, it was possible to obtain the protein content in the enzyme in milligrams of protein per gram weighed.

### 3.4. Lipase Activity

The activity of immobilized lipases was performed according to the methodology described by Cui et al. [6], with modifications. For this, the p-nitrophenol (pNF) released was evaluated, using p-nitrophenyl acetate (pNFA) as a substrate. The substrate solution was prepared with pNFA (1 mM) dissolved in isopropanol and with the addition of 0.1 g of Triton X. Control assays were performed and used to determine the background rates of p-NPA hydrolysis, based on the difference between the buffer solution and copper sulfate.

In the selected protocol, 1000 μL of phosphate buffer (100 mM and pH 7.4) was added to a 5 mg sample of nanoflower. The same protocol was used to measure the free lipase activity, with the same amounts of protein used to measure the activity of immobilized lipase and free lipase. The solution was incubated at 37 °C for 3 min at 150 revolutions per minute (rpm) to ensure temperature homogeneity in the medium. Then, 1000 μL of the substrate was added to the solution, which was again incubated and shaken for another 5 min. Finally, 10 mL of phosphate buffer was mixed into the solution, from which an aliquot of 25 μL was withdrawn, which was placed in the cuvette with 175 μL of the buffer. The reaction was carried out initially with samples of nanoflowers at pH 7.4 in different buffer concentrations (10–100 mM). Then, to better visualize the patterns of enzymatic activity, the buffer concentrations were established at different pHs (6–8).

The pNF concentration was determined using an Epoch 2 Microplate (BioTek) multiplate spectrophotometer (BioTek) at a wavelength of 400 nm, from the standard curve previously prepared for the pNF released in isopropanol. This work defines a unit of enzymatic activity as the amount of lipase that releases 1 μmol of pNF from pNFA per minute under experimental conditions. All the experiments were performed in triplicate. The hydrolytic and specific activity were calculated using Equations (1) and (2), respectively:

(1) $$HA=\frac{V\times C\;\;\;}{m\times t\;\;}\;$$

(2) $$SA=\frac{HA}{TP}$$

where HA is the hydrolytic activity (U g^−1^), C is the pNF concentration (μmol mL^−1^), V is the reaction volume (mL), m is the added mass of the biocatalyst (g), and t is the reaction time (min). SA is the specific activity (U mg^−1^) and TP is the lipase protein content (mg g^−1^). Activity (U) was defined as the amount of p-nitrophenol produced per mg of nanoflowers.

### 3.5. Computational Analysis

BCL protonation states of titratable residues were calculated using ProteinPrepare, PlayMolecule web server—playmolecule.org (accessed on 20 June 2022) [41]. The BCL structure (PDB: 2NW6) was downloaded from Protein Data Bank and uploaded into the ProteinPrepare application. The pKa calculation was performed at pHs 5 to 9, without water molecules and ligands from the input PDB file. After the calculation was performed, the protonated PDB files and protonation tables were downloaded and analyzed. The electrostatic properties were calculated using automatically configured sequential focusing multigrid calculations using the adaptive Poisson–Boltzmann solver (APBS).

The interactions between BCL with copper (Cu^2+^) was identified using the MIB web server, bioinfo.cmu.edu.tw/MIB/ (accessed on 10 July 2022) [42]. The crystal structure of BCL (PDB: 2NW6) was prepared as described in the previous section at pH 7.4 and applied for molecular docking. The complexes of BCL and Cu^2+^ were visualized and analyzed using Discovery Studio, v20 (Accelrys, San Diego, CA, USA).

### 3.6. Characterization

#### 3.6.1. Morphological Characterization of hNF-Lipase

The morphological analysis of immobilized lipase was performed using a TM 3000 Tabletop scanning electron microscope (SEM) (TM 3000, Hitachi, Tokyo, Japan) (CLQM/UFS). For the analysis was used, operating with a voltage of 15 kV, and amplifying the images from 50 to 10,000×.

The objective was to verify the morphology of the samples that formed precipitates to prove the existence of the flower conformation of hNF-lipase at different pHs (6, 7, 7.4, and 8) and phosphate buffer concentrations (25, 50, and 100 mM). The samples were placed under a microscope on carbon strips, under a vacuum, and bombarded by electron beams, interacting with the atoms in the sample. From the interactions between the electron beam and the sample, particles and radiation were produced to form an enlarged image of the sample, the micrographs.

#### 3.6.2. Physicochemical Characterization of hNF-Lipase

The physicochemical characterization of hNF-lipase was analyzed from spectra recorded by the Cary 630 FTIR spectrometer (Agilent Technologies, Waldbronn, Germany), equipped with an attenuated total reflection (ATR) mode. The analysis estimates the movement of atoms when connected through a signal obtained by the intensity of polarized light processed using Fourier transform infrared spectroscopy (FTIR) and produces the most similar infrared spectra of intensity concerning the wave number to determine the groups’ functional elements present in the interactions [43]. The peak frequencies were identified using Origin 8.5 software, to identify the characteristic peaks of free BCL and Cu_3_(PO_4_)_2_.5H_2_O compared in the hNF-lipase structure of 4000–500 cm^−1^ region.

## 4. Conclusions

Hybrid nanoflowers with lipases incorporated into their structure were synthesized at different concentrations of phosphate buffer (10–100 mM) and different pH values (6–8), demonstrating the operational stability and growth potential of these structures. At pH values 5 and 9, no precipitate formation was necessary to self-assembly of the nanostructure. This work presented, for hNF-lipase at pHs 7 and 7.4, the most relevant data concerning all the data presented, compared to more acidic or basic pHs. Despite this, pHs 6 and 8 also show evidence of nanoflower formation, with a significant increase in enzymatic activity.

The variation of hNF-lipase data under different conditions demonstrates how the loads present are influenced by changes in the process, which causes changes in the structure and enzymatic activity of each nanoflower. It was also demonstrated that the anions and cations in the solution that form the hNF-lipase are influenced by the amount of these positive or negative charges; this effect can cause the non-precipitation of the nanoparticles and even the denaturation of the enzyme. Furthermore, the presence of BCL incorporated into the structure of hNF-lipase was also confirmed.

The difference in this study lies in the computational simulation approach, which uses computational simulations to justify the behavior of the organic–inorganic complex interactions. The computational analysis was able to understand the behavior presented in the experimental data, and it was described that the possible effect of enzyme protonation on the formation of hNF-lipase occurs through the attraction and repulsion capacity of copper sulfate ions. In the present study, sites 2 and 5 are located at the ends of the enzyme lid and site 3 is close to the BCL active site region (SER87, ASP264, and HIS286). The increase in the catalytic activity of hNF-lipase was identified, compared to the free enzyme, indicating that the presence of Cu^2+^ ions in these regions of the BCL probably favors catalytic efficiency due to the active conformation of the lipase in the hNF-lipase.

Therefore, the generated nanostructures are stable and show a significant increase in enzymatic activity. The condition that exhibited the best formation was seen at pH 7.4 and the highest concentration, 100 mM, with a 20-times increase in activity concerning free BCL. The protocol still thrived in the different synthesis conditions tested, confirming the need for experimental and computational analysis strategies.

## Figures and Tables

**Table 1:** Hydrolytic activity values of free lipase and immobilized biocatalysts in the studied variations.

Biocatalysts		Hydrolytic Activity (U g^−1^)	Specific Activity (U mg^−1^)	Relative Activity (%)
BCL-free		42.62	-	100
HNF-BCL pH 7.4	10 mM	171.81	219.91	403.11
HNF-BCL pH 6	25 mM	217.34	257.33	509.96
HNF-BCL pH 7	25 mM	415.38	432.00	974.62
HNF-BCL pH 7.4	25 mM	518.00	538.07	1215.38
HNF-BCL pH 8	25 mM	182.32	188.01	427.77
HNF-BCL pH 6	50 mM	281.67	301.47	660.88
HNF-BCL pH 7	50 mM	398.42	432.39	934.81
HNF-BCL pH 7.4	50 mM	521.35	541.88	1223.26
HNF-BCL pH 8	50 mM	263.64	278.17	618.59
HNF-BCL pH 6	100 mM	362.19	410.30	849.82
HNF-BCL pH 7	100 mM	540.11	623.85	1267.27
HNF-BCL pH 7.4	100 mM	888.43	884.96	2084.96
HNF-BCL pH 8	100 mM	178.63	330.46	419.12

## Data Availability

Data are contained within the article and Supplementary Materials.

## Supplementary Materials

The following supporting information can be downloaded at: https://www.mdpi.com/article/10.3390/molecules29030628/s1, Figure S1. Molecular interaction diagrams of BCL with Cu^2+^ at binding sites with all docking score values; Table S1. Interacting amino acids predicted by MIB for BCL—Cu^2+^ complex.

## References

[1] Wu J., Wang X., Wang Q., Lou Z., Li S., Zhu Y., Qin L., Wei H. (2019). Nanomaterials with enzyme-like characteristics (nanozymes): Next-generation artificial enzymes (II). Chem. Soc. Rev..

[2] Zhong L., Feng Y., Wang G., Wang Z., Bilal M., Lv H., Jia S., Cui J. (2020). Production and use of immobilized lipases in/on nanomaterials: A review from the waste to biodiesel production. Int. J. Biol. Macromol..

[3] Li Y., Wu H., Su Z. (2020). Enzyme-based hybrid nanoflowers with high performances for biocatalytic, biomedical, and environmental applications. Coord. Chem. Rev..

[4] Yin Y., Xiao Y., Lin G., Xiao Q., Lin Z., Cai Z. (2015). An enzyme-inorganic hybrid nanoflower based immobilized enzyme reactor with enhanced enzymatic activity. J. Mater. Chem. B.

[5] Zhang M., Zhang Y., Yang C., Ma C., Tang J. (2021). Enzyme-inorganic hybrid nanoflowers: Classification, synthesis, functionalization and potential applications. Chem. Eng. J..

[6] Cui J., Zhao Y., Liu R., Zhong C., Jia S. (2016). Surfactant-activated lipase hybrid nanoflowers with enhanced enzymatic performance. Sci. Rep..

[7] de Souza R.L., de Faria E.L.P., Figueiredo R.T., Freitas L.D.S., Iglesias M., Mattedi S., Zanin G.M., Santos O.A.A.D., Coutinho J.A.P., Lima Á.S. (2013). Protic ionic liquid as additive on lipase immobilization using silica sol-gel. Enzym. Microb. Technol..

[8] Li C., Zhao J., Zhang Z., Jiang Y., Bilal M., Jiang Y., Jia S., Cui J. (2020). Self-assembly of activated lipase hybrid nanoflowers with superior activity and enhanced stability. Biochem. Eng. J..

[9] Ge J., Lei J., Zare R.N. (2012). Protein-inorganic hybrid nanoflowers. Nat. Nanotechnol..

[10] Bilal M., Asgher M., Shah S.Z.H., Iqbal H.M.N. (2019). Engineering enzyme-coupled hybrid nanoflowers: The quest for optimum performance to meet biocatalytic challenges and opportunities. Int. J. Biol. Macromol..

[11] Zhang Y., Sun W., Elfeky N.M., Wang Y., Zhao D., Zhou H., Wang J., Bao Y. (2020). Self-assembly of lipase hybrid nanoflowers with bifunctional Ca2+ for improved activity and stability. Enzym. Microb. Technol..

[12] Sharma N., Parhizkar M., Cong W., Mateti S., Kirkland M., Puri M., Sutti A. (2017). Metal ion type significantly affects the morphology but not the activity of lipase–metal–phosphate nanoflowers. RSC Adv..

[13] Altinkaynak C., Gulmez C., Atakisi O., Özdemir N. (2020). Evaluation of organic-inorganic hybrid nanoflower’s enzymatic activity in the presence of different metal ions and organic solvents. Int. J. Biol. Macromol..

[14] Liu Y., Shao X., Kong D., Li G., Li Q. (2021). Immobilization of thermophilic lipase in inorganic hybrid nanoflower through biomimetic mineralization. Colloids Surf. B Biointerfaces.

[15] Fotiadou R., Patila M., Hammami M.A., Enotiadis A., Moschovas D., Tsirka K., Spyrou K., Giannelis E.P., Avgeropoulos A., Paipetis A. (2019). Development of effective lipase-hybrid nanoflowers enriched with carbon and magnetic nanomaterials for biocatalytic transformations. Nanomaterials.

[16] Li K., Wang J., He Y., Abdulrazaq M.A., Yan Y. (2018). Carbon nanotube-lipase hybrid nanoflowers with enhanced enzyme activity and enantioselectivity. J. Biotechnol..

[17] Almeida L.C., Barbosa M.S., de Jesus F.A., Santos R.M., Fricks A.T., Freitas L.S., Pereira M.M., Lima Á.S., Soares C.M.F. (2021). Enzymatic transesterification of coconut oil by using immobilized lipase on biochar: An experimental and molecular docking study. Biotechnol. Appl. Biochem..

[18] Barbosa M.S., Freire C.C.C., Souza R.L., Cabrera-Padilla R.Y., Pereira M.M., Freire M.G., Lima Á.S., Soares C.M.F. (2019). Effects of phosphonium-based ionic liquids on the lipase activity evaluated by experimental results and molecular docking. Biotechnol. Prog..

[19] Jiang W., Wang X., Yang J., Han H., Li Q., Tang J. (2018). Lipase-inorganic hybrid nanoflower constructed through biomimetic mineralization: A new support for biodiesel synthesis. J. Colloid Interface Sci..

[20] Zhong L., Jiao X., Hu H., Shen X., Zhao J., Feng Y., Li C., Du Y., Cui J., Jia S. (2021). Activated magnetic lipase-inorganic hybrid nanoflowers: A highly active and recyclable nanobiocatalyst for biodiesel production. Renew. Energy.

[21] Ren W., Li Y., Wang J., Li L., Xu L., Wu Y., Wang Y., Fei X., Tian J. (2019). Synthesis of magnetic nanoflower immobilized lipase and its continuous catalytic application. New J. Chem..

[22] Escobar S., Velasco-Lozano S., Lu C.H., Lin Y.F., Mesa M., Bernal C., López-Gallego F. (2017). Understanding the functional properties of bio-inorganic nanoflowers as biocatalysts by deciphering the metal-binding sites of enzymes. J. Mater. Chem. B.

[23] Ke C., Fan Y., Chen Y., Xu L., Yan Y. (2016). A new lipase-inorganic hybrid nanoflower with enhanced enzyme activity. RSC Adv..

[24] Aydemir D., Gecili F., Özdemir N., Ulusu N.N. (2020). Synthesis and characterization of a triple enzyme-inorganic hybrid nanoflower (TrpE@ihNF) as a combination of three pancreatic digestive enzymes amylase, protease and lipase. J. Biosci. Bioeng..

[25] Noma S.A.A., Yılmaz B.S., Ulu A., Özdemir N., Ateş B. (2021). Development of l-asparaginase@hybrid Nanoflowers (ASNase@HNFs) Reactor System with Enhanced Enzymatic Reusability and Stability. Catal. Lett..

[26] Zheng L., Sun Y., Wang J., Huang H., Geng X., Tong Y., Wang Z. (2018). Preparation of a flower-like immobilized D-psicose 3-epimerase with enhanced catalytic performance. Catalysts.

[27] Li Y., Fei X., Liang L., Tian J., Xu L., Wang X., Wang Y. (2016). The influence of synthesis conditions on enzymatic activity of enzyme-inorganic hybrid nanoflowers. J. Mol. Catal. B Enzym..

[28] Guimarães J.R., Carballares D., Tardioli P.W., Rocha-Martin J., Fernandez-Lafuente R. (2022). Tuning Immobilized Commercial Lipase Preparations Features by Simple Treatment with Metallic Phosphate Salts. Molecules.

[29] Lisboa M.C., Rodrigues C.A., Barbosa A.S., Mattedi S., Freitas L.S., Mendes A.A., Dariva C., Franceschi E., Lima Á.S., Soares C.M.F. (2018). New perspectives on the modification of silica aerogel particles with ionic liquid used in lipase immobilization with platform in ethyl esters production. Process Biochem..

[30] Carvalho N.B., Vidal B.T., Barbosa A.S., Pereira M.M., Mattedi S., Freitas L.D.S., Lima Á.S., Soares C.M.F. (2018). Lipase immobilization on silica xerogel treated with protic ionic liquid and its application in biodiesel production from different oils. Int. J. Mol. Sci..

[31] Yu J., Wang C., Wang A., Li N., Chen X., Pei X., Zhang P., Wu S.G. (2018). Dual-cycle immobilization to reuse both enzyme and support by reblossoming enzyme-inorganic hybrid nanoflowers. RSC Adv..

[32] Kim K.K., Song H.K., Shin D.H., Kwang Y.H., Suh S.W. (1997). The crystal structure of a triacylglycerol lipase from Pseudomonas cepacia. Structure.

[33] Show P.L., Tan C.P., Anuar M.S., Ariff A., Yusof Y.A., Chen S.K., Ling T.C. (2012). Extractive fermentation for improved production and recovery of lipase derived from Burkholderia cepacia using a thermoseparating polymer in aqueous two-phase systems. Bioresour. Technol..

[34] Altinkaynak C., Yilmaz I., Koksal Z., Özdemir H., Ocsoy I., Özdemir N. (2016). Preparation of lactoperoxidase incorporated hybrid nanoflower and its excellent activity and stability. Int. J. Biol. Macromol..

[35] Barbe S., Lafaquiere V., Guieysse D., Monsan P., Remaud-Siméon M., Andre I. (2009). Insights into lid movements of Burkholderia cepacia lipase inferred from molecular dynamics simulations. Proteins Struct. Funct. Bioinform..

[36] Godoy L.C., Duquesne S., Bordes F., Sandoval G., Marty A. (2012). Lipases and phospholipases: Methods and protocols. Methods in Molecular Biology.

[37] Cui J., Jia S. (2017). Organic–inorganic hybrid nanoflowers: A novel host platform for immobilizing biomolecules. Coord. Chem. Rev..

[38] Altinkaynak C., Kocazorbaz E., Özdemir N., Zihnioglu F. (2018). Egg white hybrid nanoflower (EW-hNF) with biomimetic polyphenol oxidase reactivity: Synthesis, characterization and potential use in decolorization of synthetic dyes. Int. J. Biol. Macromol..

[39] Alhayali N.I., Ozpozan N.K., Dayan S., Ozdemir N., Yilmaz B.S. (2021). Catalase Fe3O4@Cu2+ hybrid biocatalytic nanoflowers fabrication and efficiency in the reduction of organic pollutants. Polyhedron.

[40] Bradford M.M. (1976). A Rapid and Sensitive Method for the Quantitation of Microgram Quantities of Protein Utilizing the Principle of Protein-Dye Binding. Anal. Biochem..

[41] Martinez-Rosell G., Giorgino T., de Fabritiis G., Martínez G. (2017). PlayMolecule ProteinPrepare: A Web Application for Protein Preparation for Molecular Dynamics Simulations. J. Chem. Inf. Model..

[42] Lin Y.F., Cheng C.W., Shih C.S., Hwang J.K., Yu C.S., Lu C.H. (2016). MIB: Metal Ion-Binding Site Prediction and Docking Server. J. Chem. Inf. Model..

[43] Brandao L.M.D.S., Barbosa M.S., Souza R.L., Pereira M.M., Lima Á.S., Soares C.M.F. (2021). Lipase activation by molecular bioimprinting: The role of interactions between fatty acids and enzyme active site. Biotechnol. Prog..

