# Supplementary material for: Experimental and Computational Analysis of Synthesis Conditions of Hybrid Nanoflowers for Lipase Immobilization
Source: Molecules. 2024 Jan 29;29(3):628. doi: 10.3390/molecules29030628 (PMC10856756; doi:10.3390/molecules29030628)
Supplement: Supplementary file 1 [file molecules-29-00628-s001.zip › molecules-2793591-supplementary.pdf]

Figure S1. Molecular interaction diagrams of BCL with Cu^{2+} at bind poses with all docking score values.

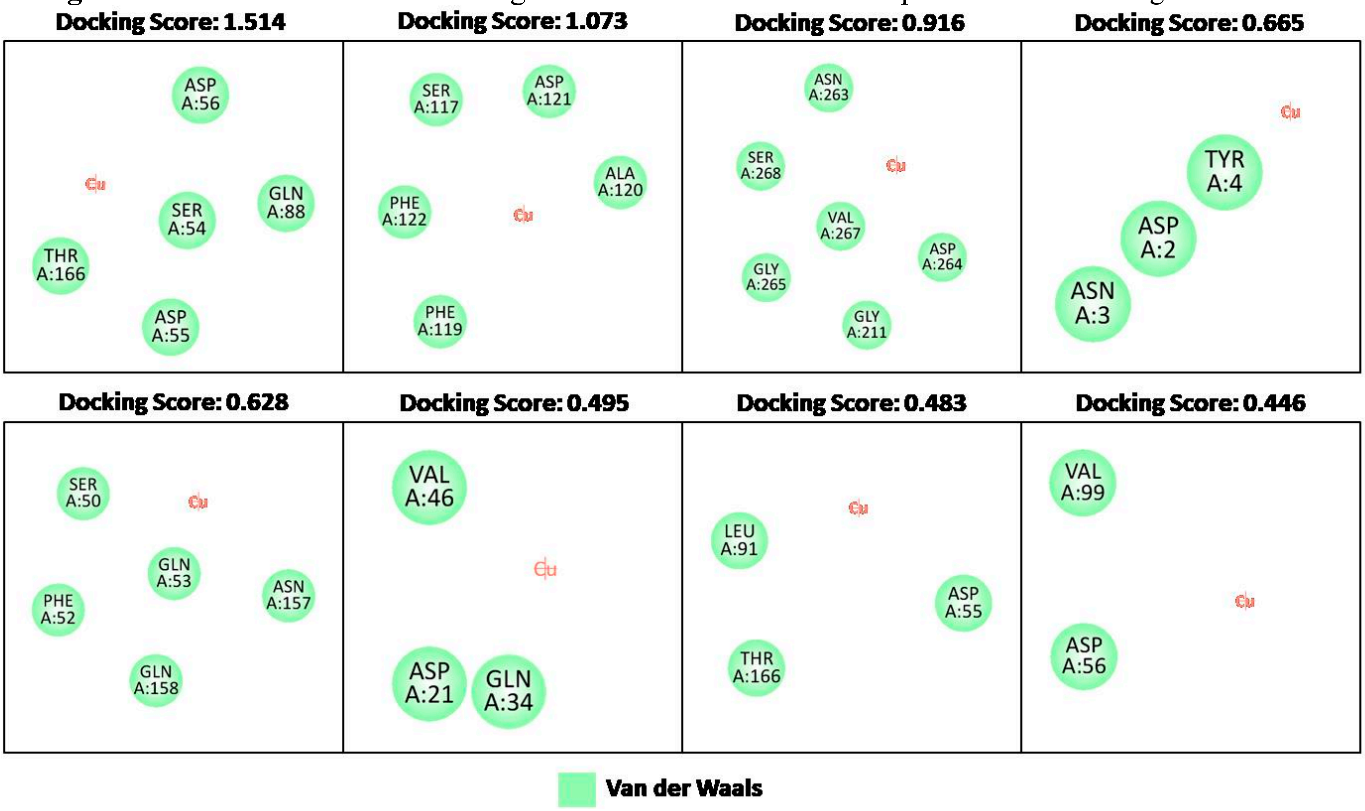

Table S1. Interacting amino acids predicted by MIB for BCL – Cu^{2+} complex.

Docking Score	Amino Acid	Type of interaction
1.514	ASP55<br>ASP56<br>GLN88<br>SER54<br>THR166	Van der Waals
1.073	*ALA120*<br>*ASP121*<br>SER117<br>*PHE122*<br>*PHE119*	Van der Waals
0.916	ASN263<br>SER268<br>VAL267<br>ASP264<br>GLY265<br>GLY211	Van der Waals
0.665	TYR4<br>ASP2<br>ASN3	Van der Waals
0.628	SER50<br>PHE52<br>GLN53<br>*ASN157*<br>*GLN158*	Van der Waals
0.495	VAL46<br>ASP21<br>GLN34	Van der Waals
0.483	LEU91<br>THR166<br>ASP55	Van der Waals
0.446	VAL99<br>ASP56	Van der Waals
0.405	-	-
